# Manipulable Electronic and Optical Properties of Two-Dimensional MoSTe/MoGe_2_N_4_ van der Waals Heterostructures

**DOI:** 10.3390/nano11123338

**Published:** 2021-12-08

**Authors:** Jiali Wang, Xiuwen Zhao, Guichao Hu, Junfeng Ren, Xiaobo Yuan

**Affiliations:** 1 School of Physics and Electronics, Shandong Normal University, Jinan 250358, China; 2020020533@stu.sdnu.edu.cn (J.W.); xwzhao@sdnu.edu.cn (X.Z.); hgc@sdnu.edu.cn (G.H.); 2 Shandong Provincial Engineering and Technical Center of Light Manipulations & Institute of Materials and Clean Energy, Shandong Normal University, Jinan 250358, China

**Keywords:** van der Waals heterostructure, electronic structure, optical absorption

## Abstract

van der Waals heterostructures (vdWHs) can exhibit novel physical properties and a wide range of applications compared with monolayer two-dimensional (2D) materials. In this work, we investigate the electronic and optical properties of MoSTe/MoGe_2_N_4_ vdWH under two different configurations using the VASP software package based on density functional theory. The results show that Te_4_-MoSTe/MoGe_2_N_4_ vdWH is a semimetal, while S_4_-MoSTe/MoGe_2_N_4_ vdWH is a direct band gap semiconductor. Compared with the two monolayers, the absorption coefficient of MoSTe/MoGe_2_N_4_ vdWH increases significantly. In addition, the electronic structure and the absorption coefficient can be manipulated by applying biaxial strains and changing interlayer distances. These studies show that MoSTe/MoGe_2_N_4_ vdWH is an excellent candidate for high-performance optoelectronic devices.

## 1. Introduction

Two-dimensional (2D) materials with different physical properties have been discovered in the last few decades, such as graphene, hexagonal boron nitride (h-BN), layered metal oxides, and transition metal dichalcogenides (TMDs) [1,2,3,4,5]. As one of the research hotspots in 2D materials, TMD materials are promising in nanoelectronics, optoelectronics, and photocatalytic devices [6,7,8,9,10]. TMD monolayers can be expressed as MX_2_, in which M represents the transition metal atom and X refers to the halogen atom. The lattice structure of TMDs is similar to a “sandwich” structure. The transition metal atom M is sandwiched between two layers of halogen atom X, which are connected by chemical bonds. One layer of the halogen atom X in MX_2_ can be replaced by another halogen atom Y, which can form a non-centrosymmetric structure and is referred to as Janus materials with the chemical formula Janus MXY [11,12]. Janus MXY have many unique physical properties that distinguish them from conventional 2D materials, including strong in-plane piezoelectric polarization [13], valley splitting [14], Rashba spin splitting [15,16], and good catalytic properties [17]. In recent years, people have conducted a lot of research on Janus MXY for M = Mo, W and X, Y = S, Se, Te, etc. As a typical representative of Janus MXY material, the Janus MoSSe monolayer has been successfully manufactured by replacing the Se atom at the top of the MoSe_2_ monolayer with the S atom, which provides experimental guidance for the synthesis of many Janus structures [17]. On the other hand, as another example, people have theoretically predicted the electronic, vibrational, elastic, and piezoelectric properties of Janus MoSTe [18]. These features prove the great potential of Janus MXY in electronic and photoelectric applications.

New 2D materials have been continuously explored, and their physical properties and potential applications have been intensively investigated. Recently, a high quality MoSi_2_N_4_ monolayer has been successfully fabricated by chemical vapor deposition (CVD) [19], which makes the new 2D material MA_2_Z_4_ family attract extensive attention, in which M = Mo, W, V, Nb, Ta, Ti, Zr, Hf, or Cr; A = Si or Ge; and Z = N, P, or As [20]. At present, people have revealed the physical properties of the MA_2_Z_4_ monolayer through first principles calculations, such as structural stability, electronic properties, visible absorption coefficient, Rashba spin splitting, carrier mobility, and so on. These findings revealed that the fascinating MA_2_Z_4_ families are promising 2D materials for many applications due to their outstanding properties [21,22,23,24,25]. Transition metal nitride (TMN) monolayers in this family, such as MoSi_2_N_4_ and MoGe_2_N_4_, are promising candidates for optoelectronic nanodevices [26,27,28,29]. Theoretical investigations have been carried out for MA_2_Z_4_ monolayer and van der Waals heterostructures (vdWH) based on it. Graphene, C_2_N, MoS_2_, and MoSe_2_ have been selected to build vdWH with MoSi_2_N_4_, which paves the way for the design of high-performance photocatalysts and optoelectronic nanodevices in the future [30,31,32,33].

vdWH, which is stacked by two or more layered materials, is one of the research hotspots of 2D materials. Compared with 2D monolayer materials, vdWH can improve electrical properties, light absorption efficiency, catalytic hydrogen production performance, etc. [34,35,36]. Although many vdWHs have been manufactured experimentally or predicted theoretically, research on 2D MA_2_Z_4_-based vdWHs is still in its infancy. Here, considering that both Janus MoSTe and MoGe_2_N_4_ monolayers are indirect band gap semiconductors [28,37] and both have good absorption coefficients in the visible region, we combine the advantages of these two monolayers and construct different configurations of MoSTe/MoGe_2_N_4_ vdWHs. The electronic structure, optical properties, and the effects of the biaxial strains and the interlayer distances are investigated. The calculation shows that a MoSTe/MoGe_2_N_4_ vdWH can be a semimetal or a direct band gap semiconductor with different stacking configurations. In addition, the optical absorption of MoSTe/MoGe_2_N_4_ vdWH has been greatly improved both in the visible and ultraviolet regions compared with those of the individual monolayers. There are transitions between semimetal and semiconductor when biaxial strains and interlayer distances are changed in MoSTe/MoGe_2_N_4_ vdWH. In addition, the compression strain enables MoSTe/MoGe_2_N_4_ vdWH to significantly increase the absorption intensity in the visible and ultraviolet regions, while the tensile strain reduces the absorption in the visible region. The construction of the MoSTe/MoGe_2_N_4_ vdWH improves the electronic structure and the optical absorption compared to its individual monolayers, and these properties can also be modulated by the strain and interlayer distance. MoSTe/MoGe_2_N_4_ vdWH can be widely used in optoelectronic nanodevices.

## 2. Calculation Methods

All our calculations are based on the first principles approach through density functional theory (DFT), which is implemented using the Vienna Ab-initio Simulation Package (VASP) [38,39]. Taking into account the exchange and other correlation energies, the general gradient approximation (GGA) [40] in the form of Perdew–Burke–Ernzerh (PBE) is used. In addition, the DFT-D3 correction method proposed by Grimme is used in the calculations [41]. For the structural optimization, electronic properties, and optical properties, the k-point in the first Brillouin zone is taken as 6 × 6 × 1 according to the Monkhorst–Pack scheme. In order to avoid the influence of interlayer interactions, a vacuum layer of 20 Å is placed perpendicular to the interface in the z-direction. During the calculation, the plane wave cut-off energy is set to 500 eV. At the same time, in order to fully optimize the atomic structure, the convergence accuracy of the force during relaxation is set to less than 0.01 eV/Å for each atom and 10^−6^ eV for the energy convergence of electron self-consistency.

Optical propagation through a medium, and the dielectric function is used to describe the absorption coefficient [42,43]. The formula is as follows:

(1) $$\varepsilon \left(\omega \right)=\varepsilon_{1}\left(\omega \right)+i\varepsilon_{2}\left(\omega \right).$$

Dielectric function *ε* is made up of two parts, real part *ε*_1_ and imaginary part *ε*_2_. The real part represents the capacity of the material to store energy, and *ε*_1_ is given by the Kramers–Kronig formula:

(2) $$\varepsilon_{1}\left(\omega \right)=1+\frac{2}{\pi }P\int_{0}^{\infty }\frac{\varepsilon_{2}^{\alpha \beta }\left(\omega^{\prime }\right)\omega^{\prime }}{{\omega^{\prime }}^{2}-\omega^{2}+i\eta }d\omega^{\prime }$$

where *P* represents the principal value of the integral.

The imaginary part *ε*_2_ represents the loss factor, which is shown in the following equation:

(3) $$\varepsilon_{2}\left(\omega \right)=\frac{4\pi^{2}e^{2}}{\Omega }\underset{q\to 0}{\lim}\underset{c:v:k}{\sum }2\omega_{k}\delta \left(\epsilon_{ck}-\epsilon_{vk}-\omega \right)\times \langle u_{ck+e_{\alpha }q}|u_{vk}\rangle \langle u_{ck+e_{\beta }q}|u_{vk}\rangle^{*}.$$

where Ω stands for the volume, *α* and *β* are the Cartesian components, *e_α_* and *e_β_* are the unit vectors, *υ* and *c* represent matrix elements of the transition from the valence band state (*u_υk_*) to the conduction band state (*u_ck_*), and *ϵ_ck_* and *ϵ_υk_* denote for the energies of the conduction and the valence band, respectively.

The absorption coefficient *α*(*ω*) is derived from the above two equations:

(4) $$\alpha \left(\omega \right)=\frac{\sqrt{2}\omega }{c}{\left\{{\left[\varepsilon_{1}^{2}\left(\omega \right)+\varepsilon_{2}^{2}\left(\omega \right)\right]}^{\frac{1}{2}}-\varepsilon_{1}\left(\omega \right)\right\}}^{\frac{1}{2}}.$$

## 3. Results and Discussions

Before studying the properties of MoSTe/MoGe_2_N_4_ vdWH, we first investigate the atomic structure and the electronic characteristics of the two monolayers. In Figure S1, both monolayers of Janus MoSTe and MoGe_2_N_4_ are semiconductors. As shown in Figure S1a, the Janus MoSTe monolayer shows a hexagonal atomic structure with the space group P6m1. The Mo atom is connected to the S and Se atoms on each side by covalent bonds. The optimized lattice parameters are a = b = 3.362 Å, the Mo-S bond length is d_Mo-s_ = 2.437 Å, and the Mo-Te bond length is d_Mo-Te_ = 2.718 Å. At the same time, the conduction band minimum (CBM) of the Janus MoSTe monolayer appears at the K point, while the valence band maximum (VBM) appears at the Γ point, and the indirect band gap is 1.027 eV. The results are consistent with previous reports [37]. The projected density of states (PDOS) of the Janus MoSTe monolayer is shown in Figure S1a. The CBM mainly comes from the strong hybridization between Mo-d, Te-p, and S-p orbitals, while the VBM is contributed from Mo-d and S-p orbitals. Similarly, the MoGe_2_N_4_ monolayer has a hexagonal crystal structure with space group P6m1. Atoms in the MoGe_2_N_4_ monolayers are connected to each other by covalent bonds in the order of N-Ge-N-Mo-N-Ge-N, so MoGe_2_N_4_ can be regarded as MoN_2_ layers sandwiched between two Ge-N bilayers. The optimized MoGe_2_N_4_ monolayers have lattice constants of a = b = 3.037 Å; the bond length of the Mo-N bond is d_Mo-N_ = 2.130 Å, and it is d_Si-N_ = 1.892 Å for the Si-N bond. As shown in Figure S1b, the MoGe_2_N_4_ monolayer is a semiconductor with an indirect band gap of 0.901 eV. Its CBM is located at the K point and is mainly contributed from Mo-d orbitals, while the VBM is located in the K-Γ path in the 2D hexagonal Brillouin zone and mainly comes from strong hybridization between Mo-d, N-p, and Ge-s orbitals. These results are consistent with the previously reported results [22].

**Figure 1:**
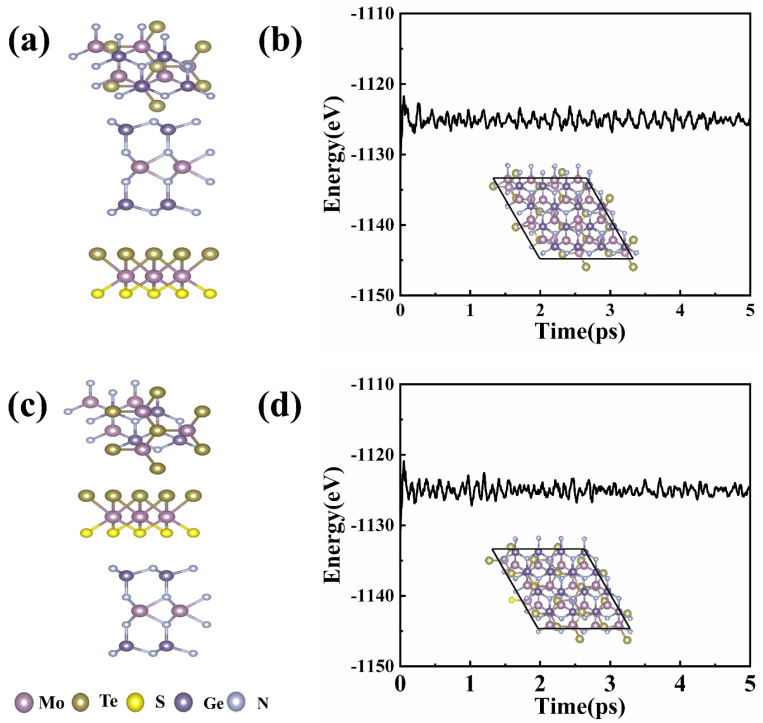
Top and side views of the optimized atomic structures of (**a**) Te_4_–MoSTe/MoGe_2_N_4_ vdWH and (**b**) S_4_–MoSTe/MoGe_2_N_4_ vdWH. The AIMD results are shown in (**c**) Te_4_–MoSTe/MoGe_2_N_4_ vdWH and (**d**) S_4_–MoSTe/MoGe_2_N_4_ vdWH, and the insets show the crystal structure at the end state.

MoSTe/MoGe_2_N_4_ vdWH is constructed by a $\sqrt{3}\times \sqrt{3}$ Janus MoSTe supercell and 2 × 2 MoGe_2_N_4_ supercell, and the lattice mismatch value is 1.48% in this case. The stacking modes are named Te_n_ vdWH when the Te atoms of Janus MoSTe are close to the MoGe_2_N_4_ layer. Similarly, the stacking method is named S_n_ vdWH when the S atom of Janus MoSTe is close to the MoGe_2_N_4_ layer. There are six possible stacking configurations for Te_n_ and S_n_, respectively, so there are twelve different stacking configurations, which are shown in Figure S2. To check the stability of these different stacking configurations, we calculate their binding energies. The binding energy can be expressed as $E_{b}=E_{\mathrm{total}}-E_{\mathrm{MoSTe}}-E_{\mathrm{MoGe}2N4}$, where *E*_total_, *E*_MoSTe_, and *E*_MoGe2N4_ are the total energies of the MoSTe/MoGe_2_N_4_ vdWH, the Janus MoSTe, and the MoGe_2_N_4_ monolayer, respectively. The binding energies for the twelve stacking configurations are listed in Table S1. All binding energies are negative, which indicates that the twelve stacking configurations are stable. The binding energies of Te_4_ and S_4_ are lowest compared with the others, which means that these two stacking configurations are the most stable. Therefore, in the subsequent calculations, we focus on Te_4_ and S_4_, which are shown in Figure 1. We calculate the ab initio molecular dynamics (AIMD) of Te_4_ and S_4_ at 300 K with a simulation duration of 5 ps and a time step of 1 fs. As shown in Figure 1, it is found that the fluctuation of energies is small, which indicates that the two configurations are stable.

**Figure 2:**
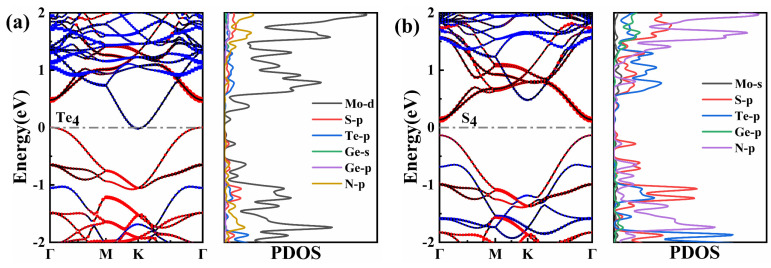
The weighted band structures and PDOS of (**a**) Te_4_–MoSTe/MoGe_2_N_4_ vdWH and (**b**) S_4_–MoSTe/MoGe_2_N_4_ vdWH. The Fermi levels are set to zero. Red represents the weights by Janus MoSTe; blue indicates weights by MoGe_2_N_4_.

As shown in Figure 2, based on the orbital characteristics, we plot the energy band structure and the PDOS of the vdWH using different color plots. It can be found that different stacking patterns can produce different electronic structures due to different interlayer coupling effects. Figure 2a shows the Te_4_-MoSTe/MoGe_2_N_4_ stacked conformation. There is no band gap in Te_4_-MoSTe/MoGe_2_N_4_ vdWH. Its CBM is located at point Γ, mainly coming from the contribution of the MoGe_2_N_4_ monolayer, and VBM is located at point K, mainly coming from the contribution of the Janus MoSTe monolayer. This vdWH has a non-overlapping band gap between the two semiconductors, which is crossed through the vdWH by band-to-band quantum tunneling and charge transfer. Therefore, the Te_4_-MoSTe/MoGe_2_N_4_ vdWH is a semimetal, which can be widely used in the design of various high-speed and low-power devices [44]. It can be seen from PDOS that CBM mainly comes from N-p orbitals and VBM mainly comes from S-p orbitals. Figure 2b shows the S_4_-MoSTe/MoGe_2_N_4_ vdWH stacked configuration, and it is clear that S_4_-MoSTe/MoGe_2_N_4_ vdWH has a direct band gap of 0.27 eV, where both CBM and VBM are located at the Γ point. It can be seen that the CBM and VBM come from the contributions of the Janus MoSTe monolayer and the MoGe_2_N_4_ monolayer, respectively. It can be seen from the PDOS that the CBM is mainly from the Mo-s orbital and the VBM is mainly from the S-p orbital. This direct band gap semiconductor of S_4_-MoSTe/MoGe_2_N_4_ vdWH can effectively facilitate the separation of electrons and holes in real space. Therefore, this finding makes MoSTe/MoGe_2_N_4_ vdWH a promising candidate for the fabrication of high-performance optoelectronic devices with suppressed carrier complexation.

**Figure 3:**
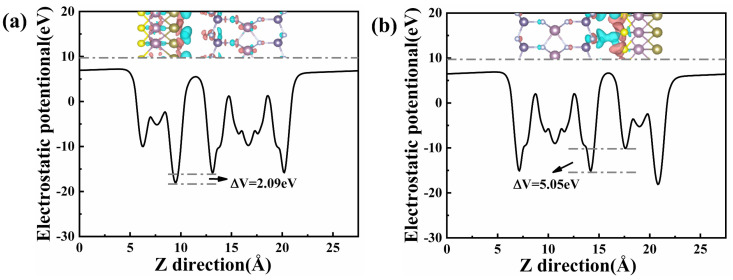
The electrostatic potential diagram in (**a**) Te_4_–MoSTe/MoGe_2_N_4_ vdWH and (**b**) S_4_–MoSTe/MoGe_2_N_4_ vdWH. The top inset shows the differential charge density. The pink and blue colors indicate electron accumulation and loss, respectively. The isosurface value is 0.0002 eV Å^−1^.

Figure 3 shows the electrostatic potential and differential charge density for two stacked configurations of MoSTe/MoGe_2_N_4_ vdWH. The electrostatic potential can reflect the difficulty of the electron transport system and has an impact on the electrical properties of the material. As can be seen from Figure 3, the electrostatic potentials of the two stacking configurations are slightly different. The electrostatic potential of the Janus MoSTe monolayer is deeper than that of the MoGe_2_N_4_ monolayer. The electrostatic potential of Te_4_-MoSTe/MoGe_2_N_4_ vdWH decreases by 2.09 eV, while the electrostatic potential of S_4_-MoSTe/MoGe_2_N_4_ vdWH decreases by 5.05 eV. Therefore, there is a built-in electric field between the two layers with the direction from the Janus MoSTe layer to the MoGe_2_N_4_ layer, which affects the charge injection and the carrier motion to some extent.

In order to visualize the charge transfer of MoSTe/MoGe_2_N_4_ vdWH, we calculate the charge density difference as follows:

(5) $$\Delta \rho =\rho_{\mathrm{MoSTe}/\mathrm{MoGe}2N4}-\rho_{\mathrm{MoSTe}}-\rho_{\mathrm{MoGe}2N4}$$

where *ρ*_MoSTe/MoGe2N4_, *ρ*_MoSTe_, and *ρ*_MoGe2N4_ denote the charge density of MoSTe/MoGe_2_N_4_ vdWH, Janus MoSTe, and MoGe_2_N_4_ monolayer, respectively. In Figure 3, the pink represents electron accumulation and blue represents electron depletion. For Te_4_-MoSTe/MoGe_2_N_4_ vdWH, the electrons are gathered around the Mo atoms in Janus MoSTe and MoGe_2_N_4_ layers, and there is no electron transfer between the two layers, as shown in Figure 3a. From Figure 3b, we can clearly see the electron distribution between the two monolayers in S_4_-MoSTe/MoGe_2_N_4_ vdWH. We observe that the electron accumulates in the Janus MoSTe layer, while there is an electron depletion in the MoGe_2_N_4_ layer. This shows that the electron is transferred from the MoGe_2_N_4_ layer to the Janus MoSTe layer. By Bader charge analysis, we found that only a small amount of 0.575 eV electrons are transferred from the Janus MoSTe layer to the MoGe_2_N_4_ layer.

**Figure 4:**
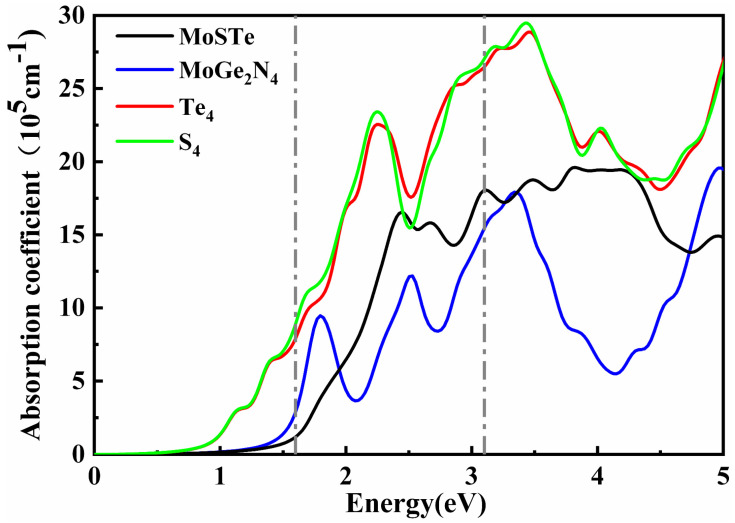
Optical absorption spectrum of Janus MoSTe, MoGe_2_N_4,_ Te_4_–MoSTe/MoGe_2_N_4_ vdWH, and S_4_–MoSTe/MoGe_2_N_4_ vdWH.

Figure 4 shows the calculated optical absorption spectrum of Janus MoSTe, MoGe_2_N_4,_ Te_4_-MoSTe/MoGe_2_N_4_ vdWH, and S_4_-MoSTe/MoGe_2_N_4_ vdWH. In the visible region (1.6 eV < E < 3.1 eV), both the Janus MoSTe monolayer and MoGe_2_N_4_ monolayer have a high absorption coefficient. Combining the advantages of the two monolayers, the absorption coefficient is significantly increased after the vdWH is formed. Compared with the two independent monolayers, both Te_4_-MoSTe/MoGe_2_N_4_ vdWH and S_4_-MoSTe/MoGe_2_N_4_ vdWH show stronger absorption coefficients. In the ultraviolet (UV) region, the absorption intensity has a maximum. Due to interlayer coupling between Janus MoSTe and MoGe_2_N_4_ monolayers, the band gap of MoSTe/MoGe_2_N_4_ vdWH is reduced and charge transfer takes place, resulting in an enhanced absorption spectrum. Thus, MoSTe/MoGe_2_N_4_ vdWH has a higher optical response and is more favorable for applications in optoelectronic devices compared with monolayers.

**Figure 5:**
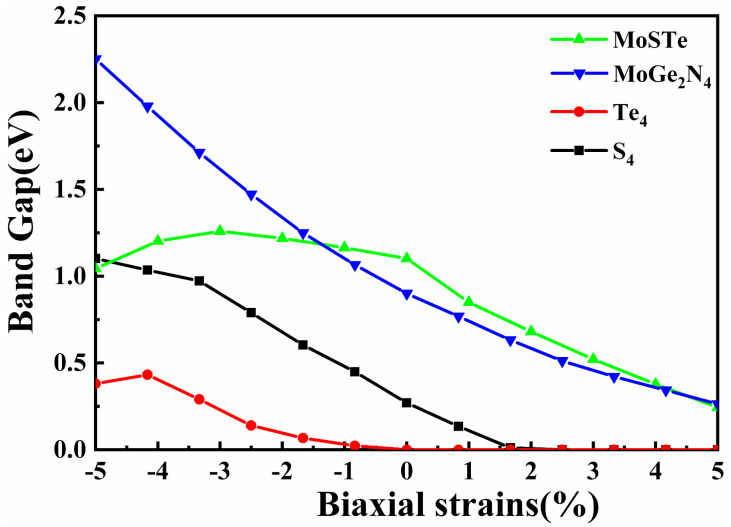
Effect of applied biaxial strain on band gap for Janus MoSTe, MoGe_2_N_4,_ Te_4_–MoSTe/MoGe_2_N_4_ vdWH, and S_4_–MoSTe/MoGe_2_N_4_ vdWH, respectively.

Thus far, researchers have found that by applying external stress, it is possible not only to change the electronic properties of two-dimensional materials but also to achieve modulation of the absorption coefficient. In the following study, biaxial strains are applied to Te_4_-MoSTe/MoGe_2_N_4_ vdWH and S_4_-MoSTe/MoGe_2_N_4_ vdWH, and both their electronic and optical properties are investigated. The equations for calculating the strain magnitude of the materials are as follows [45]: $\varepsilon =\left[\left(a-a_{0}\right)/a_{0}\right]\times 100\%$, where *a* and *a*_0_ represent the lattice constants for applied and unapplied stress, respectively. *ε* < 0 means compressive stress, and *ε* > 0 means tensile stress. Stress of −5% ≤ *ε* ≤ 5% is applied to Te_4_-MoSTe/MoGe_2_N_4_ vdWH and S_4_-MoSTe/MoGe_2_N_4_ vdWH, respectively. The variation of band gap with biaxial strain for the Janus MoSTe monolayer, MoGe_2_N_4_ monolayer, and MoSTe/MoGe_2_N_4_ vdWH is shown in Figure 5.

The formed MoSTe/MoGe_2_N_4_ vdWH has a significantly smaller band gap compared with those of the Janus MoSTe and the MoGe_2_N_4_ monolayer. Te_4_-MoSTe/MoGe_2_N_4_ vdWH still shows metallicity, and there is no band gap when tensile strain is applied. However, with increasing compressive strain, the band gap of Te_4_-MoSTe/MoGe_2_N_4_ vdWH gradually increases from zero, and the electronic structure changes from semimetal to semiconductor. When the compressive strain is greater than −5%, the band gap starts to decrease again. At this time, its CBM and VBM are coming from the MoGe_2_N_4_ monolayer and Janus MoSTe monolayer, respectively, as shown in Figure S3. The band gap of S_4_-MoSTe/MoGe_2_N_4_ vdWH increases with increasing compressive strain and decreases with increasing tensile strain. However, when a biaxial strain larger than +2% is applied, there is no band gap and S_4_-MoSTe/MoGe_2_N_4_ vdWH becomes a semimetal. Therefore, the electronic structure of MoSTe/MoGe_2_N_4_ vdWH can be regulated to change between semimetal and semiconductor by applying biaxial strain.

**Figure 6:**
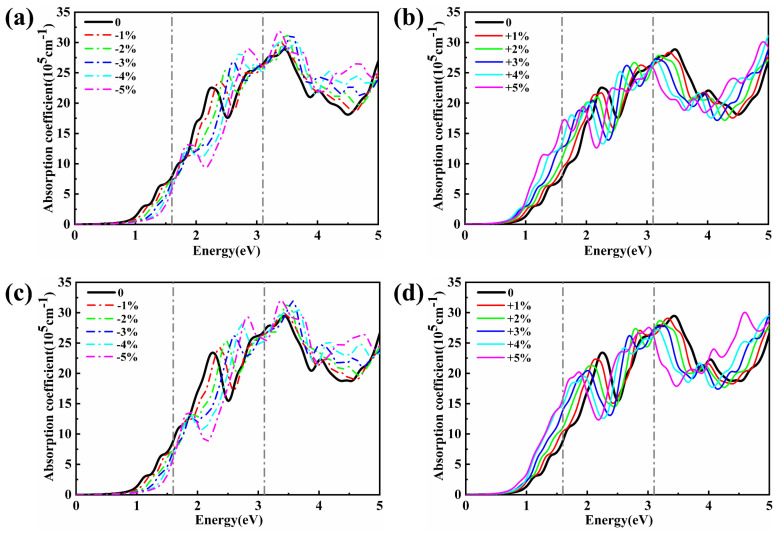
Optical absorption spectrum of (**a**,**b**) Te_4_–MoSTe/MoGe_2_N_4_ vdWH and (**c**,**d**) S_4_–MoSTe/MoGe_2_N_4_ vdWH under biaxial strain. Negative and positive values represent compressive stress and tensile stress, respectively.

The optical properties of MoSTe/MoGe_2_N_4_ vdWH also change after the biaxial strain is applied. As shown in Figure 6, we investigate the variation patterns of the absorption coefficients of Te_4_-MoSTe/MoGe_2_N_4_ vdWH and S_4_-MoSTe/MoGe_2_N_4_ vdWH with biaxial strain. It is found that the effects of biaxial strain on the absorption intensity of the two vdWHs are basically the same pattern. When compressive strain is applied, the absorption coefficient of MoSTe/MoGe_2_N_4_ vdWH decreased with increasing compressive intensity and increased with increasing tensile intensity in the infrared region. In the visible and UV regions, the absorption coefficient of MoSTe/MoGe_2_N_4_ vdWH increases with increasing compressive strength and decreases with increasing tensile strength. It can be seen that compressive strain increases the absorption coefficient of MoSTe/MoGe_2_N_4_ vdWH in the visible and UV regions, while tensile strain increases the absorption coefficient of the vdWH in the infrared region. Therefore, biaxial strain can also effectively manipulate the absorption properties of MoSTe/MoGe_2_N_4_ vdWH.

**Figure 7:**
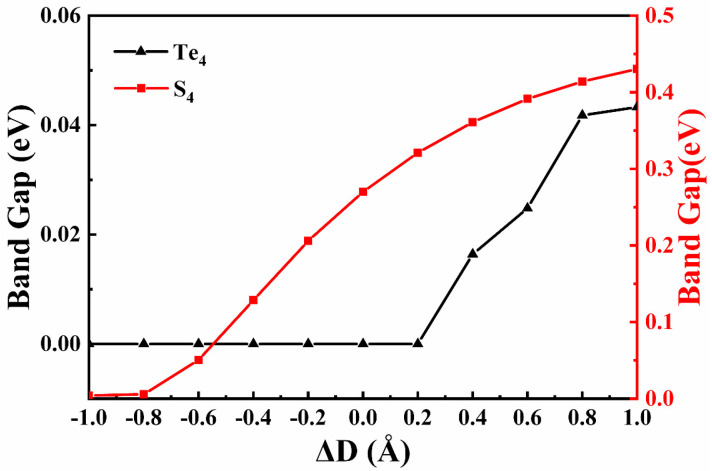
Variations in band gaps of the Te_4_–MoSTe/MoGe_2_N_4_ vdWH and S_4_–MoSTe/MoGe_2_N_4_ vdWH with the change in interlayer distances. Black and red correspond to Te_4_–MoSTe/MoGe_2_N_4_ vdWH and S_4_–MoSTe/MoGe_2_N_4_ vdWH, respectively.

The variations of the band gaps with different interlayer distances for Te_4_-MoSTe/MoGe_2_N_4_ and S_4_-MoSTe/MoGe_2_N_4_ vdWH are shown in Figure 7, where the equilibrium interlayer distances are 3.4 Å and 3.1 Å, respectively. We find that there is no band gap in Te_4_-MoSTe/MoGe_2_N_4_ vdWH when the interlayer distance is less than 3.63 Å. When the interlayer distance is larger than 3.63 Å, the band gap of Te_4_-MoSTe/MoGe_2_N_4_ vdWH increases with an increase in the interlayer distance. The Te_4_-MoSTe/MoGe_2_N_4_ vdWH changes between semimetal and indirect band gap semiconductors when the interlayer distance changes. The weight band structures of Te_4_-MoSTe/MoGe_2_N_4_ and S_4_-MoSTe/MoGe_2_N_4_ vdWH at different interlayer distances are depicted in Figure S4. We find that the band gap in S_4_-MoSTe/MoGe_2_N_4_ vdWH increases with an increasing interlayer distance and decreases with a decreasing interlayer distance. When the interlayer distance is less than 2.3 Å, the band gap of the S_4_-MoSTe/MoGe_2_N_4_ vdWH becomes small and close to zero. The results indicate that changing the interlayer distance can also regulate the electronic structure of the MoSTe/MoGe_2_N_4_ vdWH.

## 4. Conclusions

In conclusion, we systematically researched the atomic structure, electronic properties, and optical properties of MoSTe/MoGe_2_N_4_ vdWH using the VASP software package based on density functional theory. Two monolayers of Janus MoSTe and MoGe_2_N_4_ are stacked into heterostructures by van der Waals forces. The thermodynamic stability of MoSTe/MoGe_2_N_4_ vdWH is examined using AIMD simulations. Two stacking modes have low binding energies, i.e., Te_4_-MoSTe/MoGe_2_N_4_ vdWH and S_4_-MoSTe/MoGe_2_N_4_ vdWH. Compared with the two separate monolayers, the band gap of MoSTe/MoGe_2_N_4_ vdWH is reduced and the light absorption intensity is significantly increased. The energy band structure of MoSTe/MoGe_2_N_4_ vdWH can be manipulated by applying a biaxial strain, and the transition between semimetal and semiconductor appears when the biaxial strain changes from −5% to +5%. At the same time, the applied strain can also modulate the light absorption intensity. In the visible and UV regions, the absorption coefficient increases with increasing compressive strain and decreases with increasing tensile strain. In addition, different interlayer distances contribute to a change in the band gap, and a shift between semiconductor and semimetal can be realized. In other words, the electronic and optical properties of MoSTe/MoGe_2_N_4_ vdWH can be manipulated by external conditions, such as applying external strain and changing the interlayer distances. Our research has shown that MoSTe/MoGe_2_N_4_ vdWH is a potential candidate for high-performance optoelectronic nanodevices.

## Data Availability

The data are available on request from the corresponding author.

## Supplementary Materials

The following are available online at https://www.mdpi.com/article/10.3390/nano11123338/s1, Figure S1: Band structures and PDOS of (a) Janus MoSTe monolayer and (b) MoGe_2_N_4_ monolayer. The Fermi levels are set to zero, Figure S2: The optimized top and side views of MoSTe/MoGe_2_N_4_ vdWH for twelve different stacking patterns, Figure S3: Band structures of (a) (b) Te_4_-MoSTe/MoGe_2_N_4_ vdWH and (c) (d) S_4_-MoSTe/MoGe_2_N_4_ vdWH under biaxial strain. Red and blue represent the weights from Janus MoSTe and MoGe_2_N_4_ monolayers, respectively. Negative and positive values mean compressive stress and tensile stress, Figure S4: Band structures of (a) (b) Te_4_-MoSTe/MoGe_2_N_4_ vdWH and (c) (d) S_4_-MoSTe/MoGe_2_N_4_ vdWH under different interlayer distances, where the equilibrium interlayer distances are 3.4 Å and 3.1Å, respectively. Red and blue represent the weights from Janus MoSTe and MoGe_2_N_4_ monolayers, respectively, Table S1: Binding energies (E_b_) of the optimized MoSTe/MoGe_2_N_4_ vdWH for the twelve different stacking patterns.
