# Supplementary material for: Manipulable Electronic and Optical Properties of Two-Dimensional MoSTe/MoGe2N4 van der Waals Heterostructures
Source: Nanomaterials (Basel). 2021 Dec 8;11(12):3338. doi: 10.3390/nano11123338 (PMC8709393; doi:10.3390/nano11123338)
Supplement: Supplementary file 1 [file nanomaterials-11-03338-s001.zip › nanomaterials-1473357-supplementary.pdf]

nanomaterials

MDPI

Supplementary Materials

Manipulable Electronic and Optical Properties of Two-Dimensional $MoSTe/MoGe_2N_4$ van der Waals Heterostructures

Jiali Wang [1], Xiuwen Zhao [1], Guichao Hu [1], Junfeng Ren [1,2,*] and Xiaobo Yuan [1,*]

[1] School of Physics and Electronics, Shandong Normal University, Jinan 250358, China; 2020020533@stu.sdnu.edu.cn (J.W.); xwzhao@sdnu.edu.cn (X.Z.); hgc@sdnu.edu.cn (G.H.)
[2] Shandong Provincial Engineering and Technical Center of Light Manipulations & Institute of Materials and Clean Energy, Shandong Normal University, Jinan 250358, China
* Correspondence: renjf@sdnu.edu.cn (J.R.); yxb@sdnu.edu.cn (X.Y.)

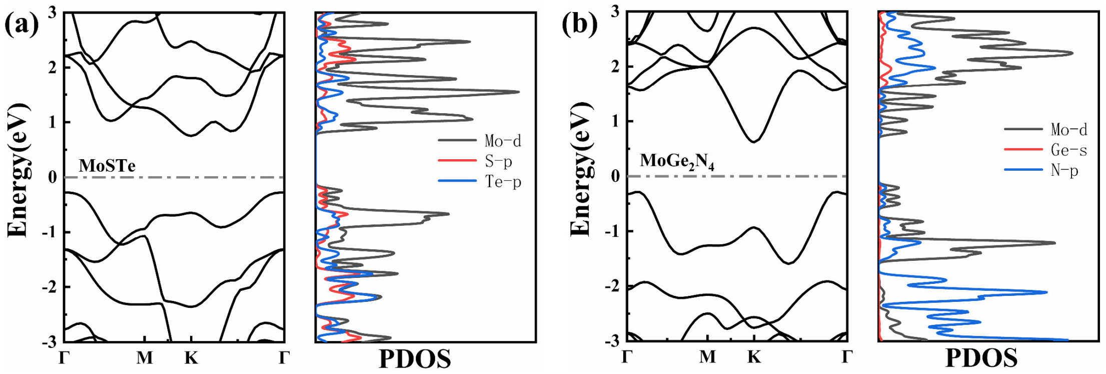

Figure S1. Band structures and PDOS of (**a**) Janus MoSTe monolayer and (**b**) $MoGe_2N_4$ monolayer, respectively. The Fermi levels are set to zero.

Table S1. : Binding energies (E_b) of the optimized $MoSTe/MoGe_2N_4$ vdWH for the twelve different stacking patterns.

Heterostructure	Te_1	Te_2	Te_3	Te_4	Te_5	Te_6
E_b (meV)	-6.7680	-6.7679	-6.7671	-6.7710	-6.7670	-6.7708

Heterostructure	S_1	S_2	S_3	S_4	S_5	S_6
E_b (meV)	-6.7396	-6.7407	-6.7405	-6.7434	-6.7427	-6.7394

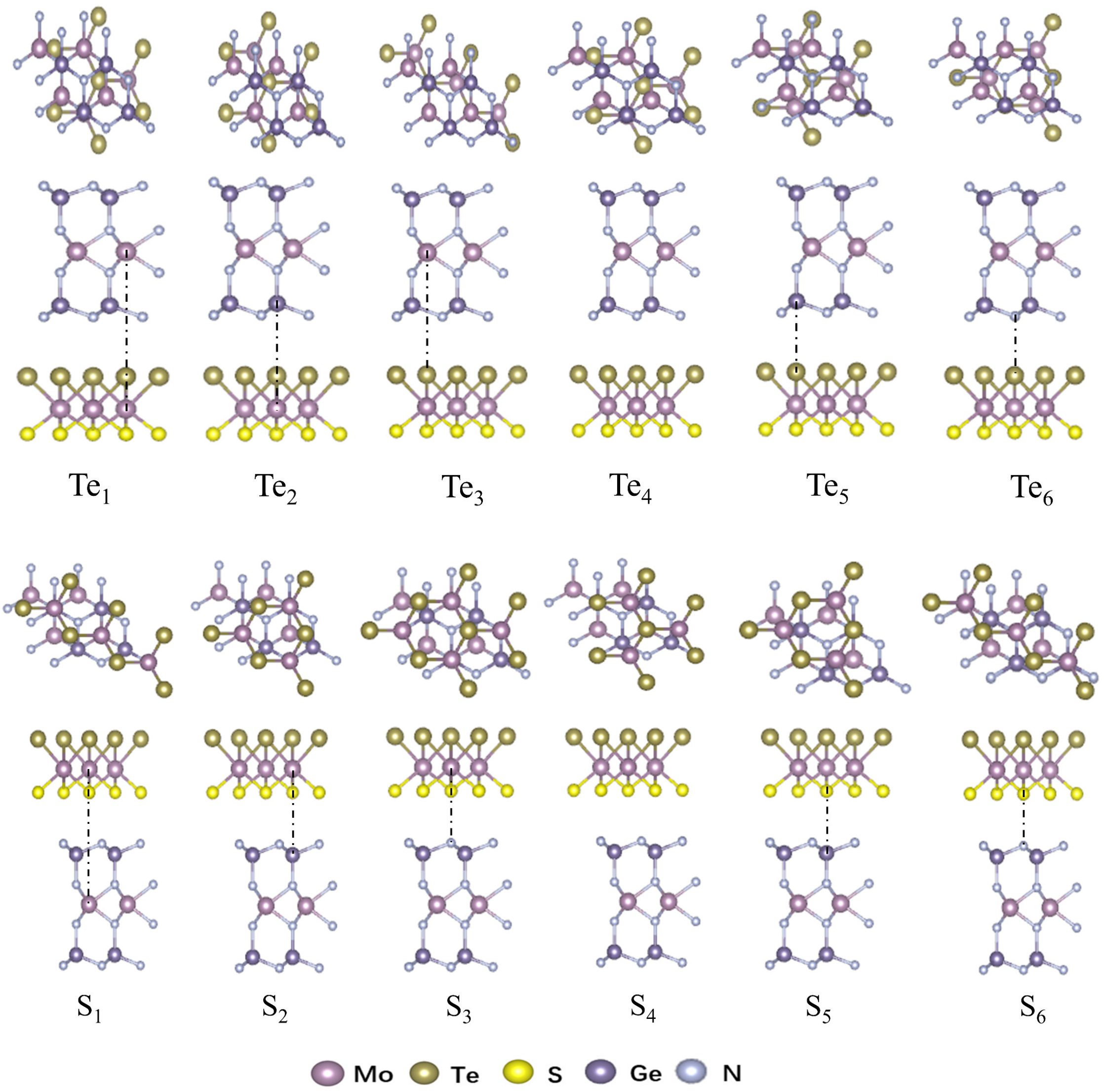

Figure S2. The optimized top and side views of the $MoSTe/MoGe_2N_4$ vdWH for twelve different stacking patterns.

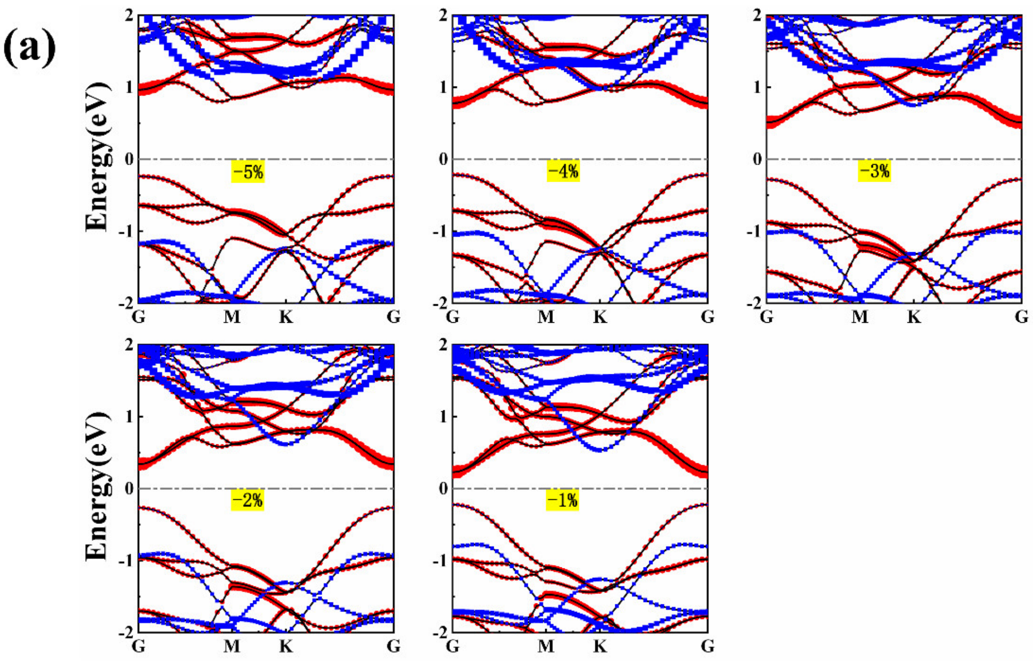

(b)

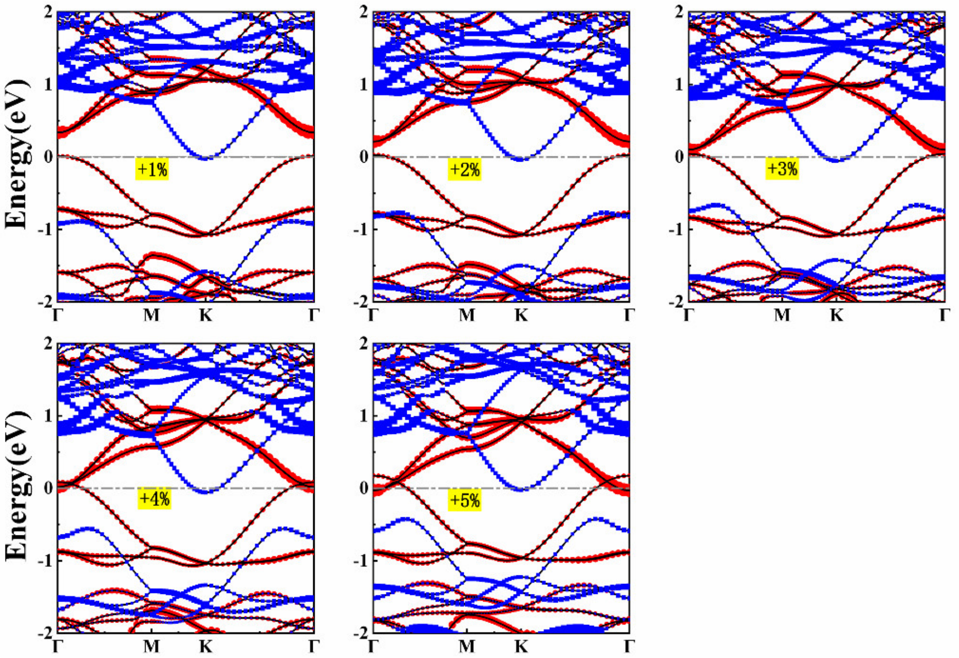

(c)

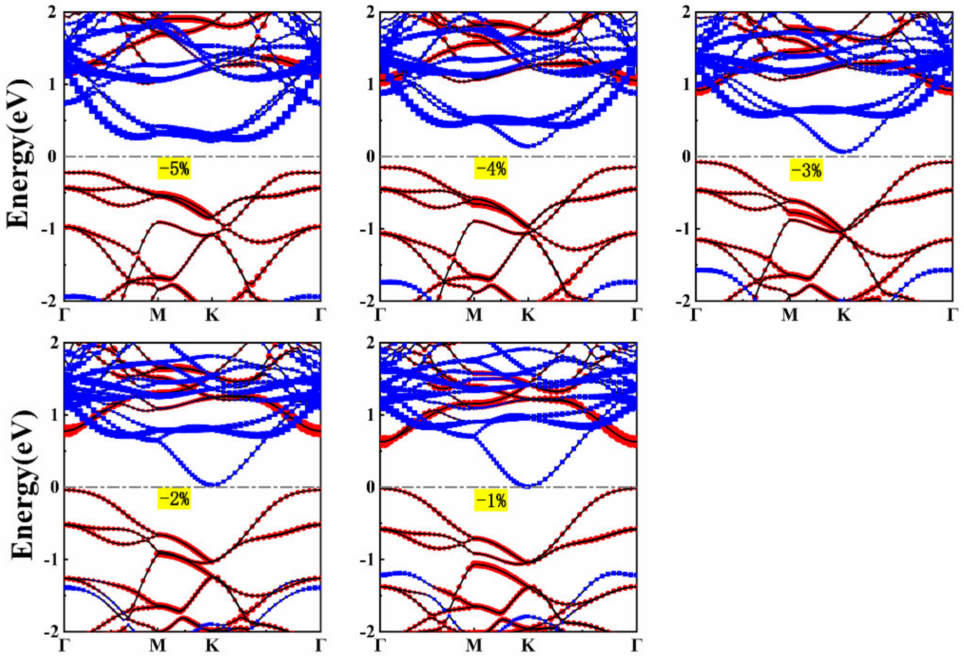

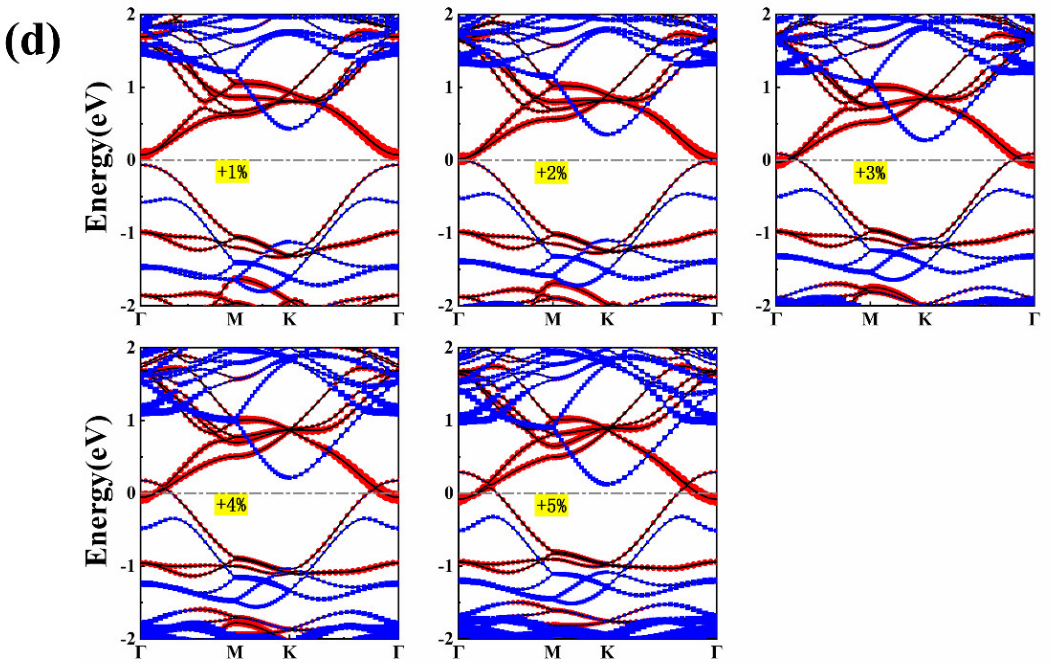

Figure S3. Band structures of (**a**) (**b**) Te_4-MoSTe/$MoGe_2N_4$ vdWH, (**c**) (**d**) S_4-MoSTe/$MoGe_2N_4$ vdWH under biaxial strains. The red and blue represent the weights from Janus MoSTe and $MoGe_2N_4$ monolayers, respectively. Negative and positive values mean compressive stress and tensile stress.

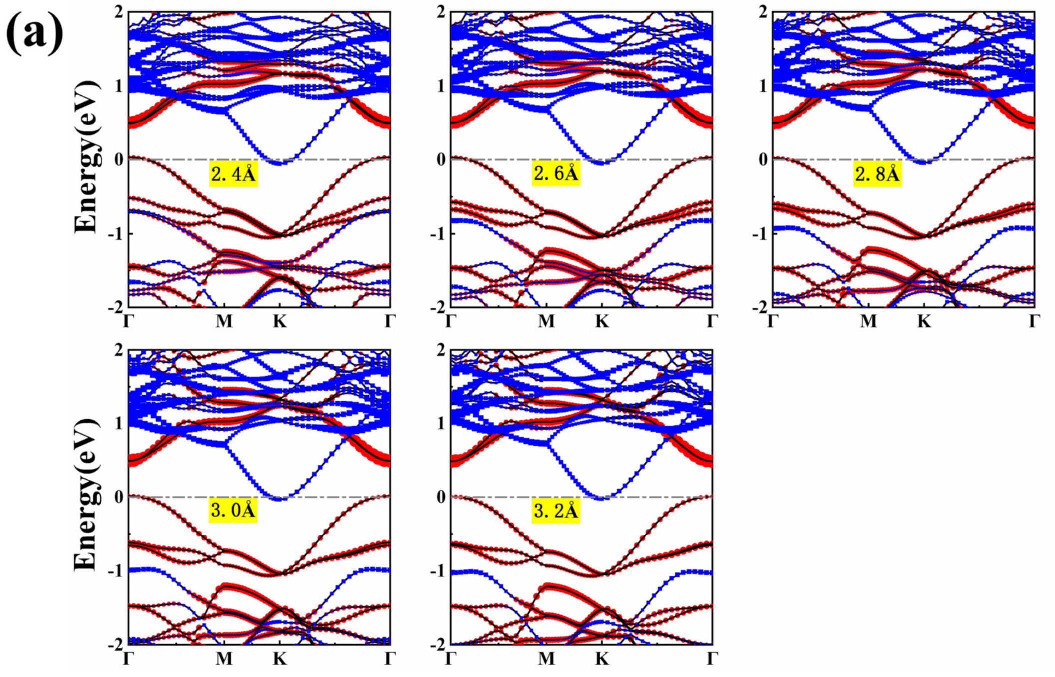

(b)

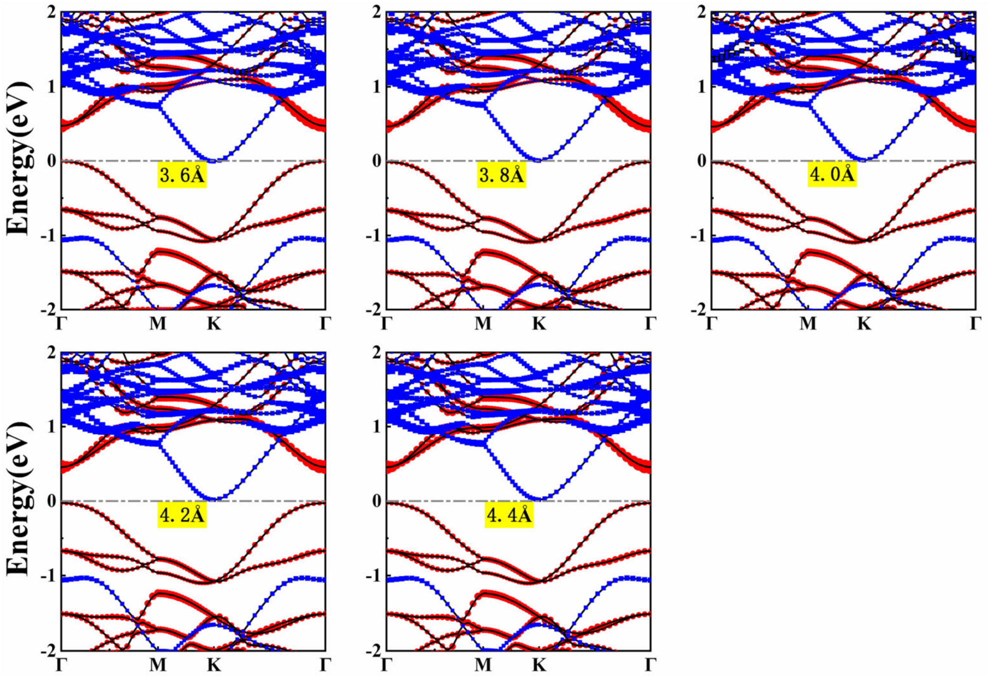

Energy(eV)
2
1
0
-1
-2
Γ
M
K
Γ
3. 6Å
3. 8Å
4. 0Å
4. 2Å
4. 4Å

(c)

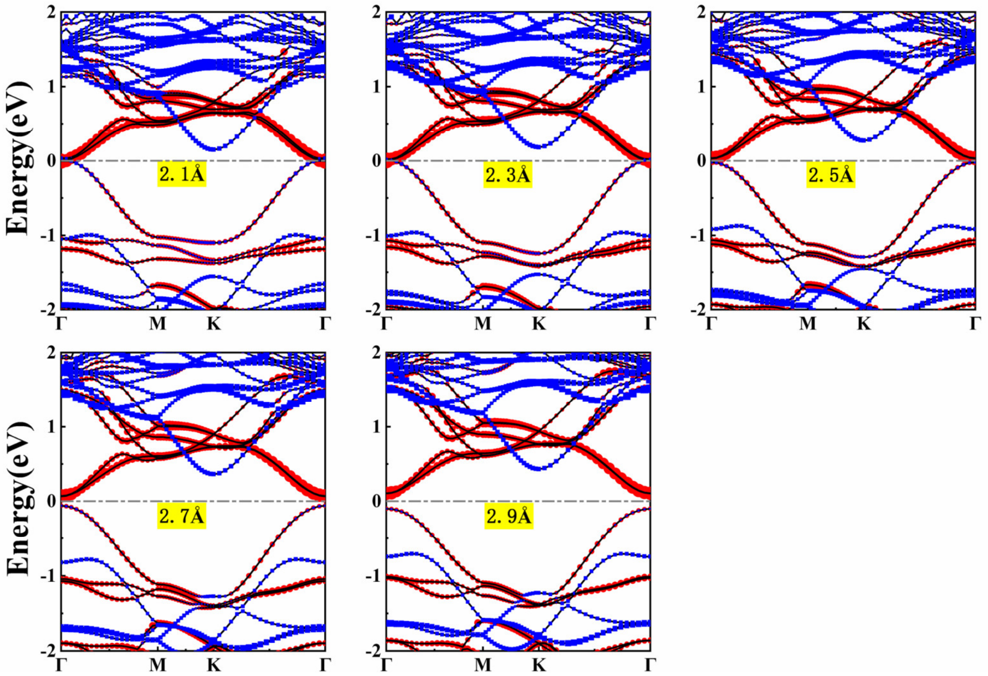

Energy(eV)
2
1
0
-1
-2
Γ
M
K
Γ
2. 1Å
2. 3Å
2. 5Å
2. 7Å
2. 9Å

(d)

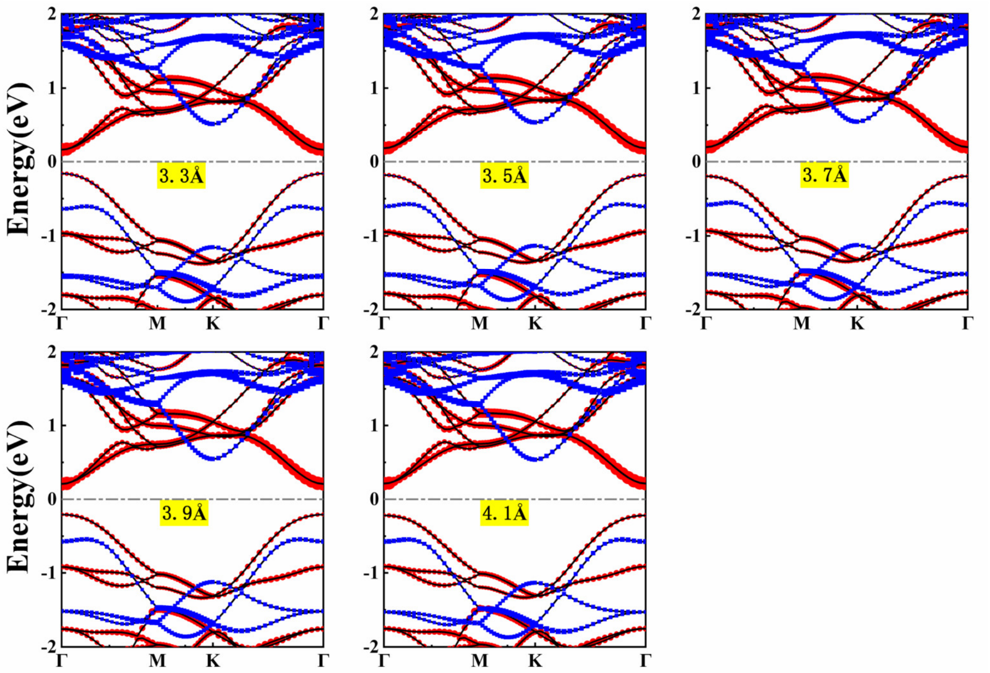

Figure S4. Band structures of (**a**) (**b**) Te_4-MoSTe/$MoGe_2N_4$ vdWH, (**c**) (**d**) S_4-MoSTe/$MoGe_2N_4$ vdWH under different interlayer distances, where the equilibrium interlayer distances are 3.4 Å and 3.1Å, respectively. The red and blue represent the weights from Janus MoSTe and $MoGe_2N_4$ monolayers, respectively. .
